# BIA-ALCL and BIA-SCC: Updates on Clinical Features and Genetic Mutations for Latest Recommendations

**DOI:** 10.3390/medicina60050793

**Published:** 2024-05-10

**Authors:** Gennaro D’Orsi, Martina Giacalone, Alessio Calicchia, Elettra Gagliano, Lisa Vannucchi, Gianluca Vanni, Oreste Claudio Buonomo, Valerio Cervelli, Benedetto Longo

**Affiliations:** 1 PhD School of Applied Medical-Surgical Sciences, Tor Vergata University of Rome, Via Montpellier 1, 00133 Rome, Italy; 2 Plastic and Reconstructive Surgery at Department of Surgical Science, Tor Vergata University of Rome, Via Montpellier 1, 00133 Rome, Italy; 3 Division of Breast Unit, Department of Surgical Sciences, School of Medicine and Surgery, Tor Vergata University of Rome, Via Montpellier 1, 00133 Rome, Italy

**Keywords:** BIA-ALCL, BIA-SCC, breast implants, breast reconstruction

## Abstract

Breast Implant-Associated Anaplastic Large Cell Lymphoma (BIA-ALCL) and Breast Implant-Associated Squamous Cell Carcinoma (BIA-SCC) are emerging neoplastic complications related to breast implants. While BIA-ALCL is often linked to macrotextured implants, current evidence does not suggest an implant-type association for BIA-SCC. Chronic inflammation and genetics have been hypothesized as key pathogenetic players, although for both conditions, the exact mechanisms and specific risks related to breast implants are yet to be established. While the genetic alterations in BIA-SCC are still unknown, JAK-STAT pathway activation has been outlined as a dominant signature of BIA-ALCL. Recent genetic investigation has uncovered various molecular players, including MEK-ERK, PI3K/AKT, CDK4-6, and PDL1. The clinical presentation of BIA-ALCL and BIA-SCC overlaps, including most commonly late seroma and breast swelling, warranting ultrasound and cytological examinations, which are the first recommended steps as part of the diagnostic work-up. While the role of mammography is still limited, MRI and CT-PET are recommended according to the clinical presentation and for disease staging. To date, the mainstay of treatment for BIA-ALCL and BIA-SCC is implant removal with en-bloc capsulectomy. Chemotherapy and radiation therapy have also been used for advanced-stage BIA-ALCL and BIA-SCC. In-depth characterization of the tumor genetics is key for the development of novel therapeutic strategies, especially for advanced stage BIA-ALCL and BIA-SCC, which show a more aggressive course and poor prognosis.

## 1. Introduction

Nowadays, breast implants are in common use for both reconstruction purposes following mastectomy and aesthetic breast augmentation or remodeling. Despite initial concerns, several studies have shown that breast implants do not increase the risk of breast tissue cancer [1]. Nonetheless, the past decades have made the risk of breast implant capsule-associated tumors ever more evident [2]. The first report on Breast Implant-Associated Anaplastic Large Cell Lymphoma (BIA-ALCL) dates to 1997 when Keech and Creech first described the occurrence of a type of anaplastic T-cell lymphoma around breast implants, recognized in 2016 by the WHO as a new type of lymphoma arising from the breast implant capsule [3]. In 1994, Kitchen reported the first case of Breast Implant-associated Squamous Cell Carcinoma (BIA-SCC) in a patient who had undergone breast augmentation [4]. As of today, BIA-ALCL is the most frequent among breast implant-associated tumors, while only a few cases of breast implant-associated squamous cell carcinoma (BIA-SCC) and B-cell lymphomas of the implant capsule have been described [5,6]. Despite being rare tumors overall, an increasing number of reports have urged the attention of the medical community and competent authorities on medical device safety. 

Current evidence suggests that breast implants—of different types and textures—may favor alterations on nearby tissues and cells recruited at the implant capsule, which, in the long-term and under given stimuli, might cause tumorigenesis [7]. Despite the investigation conducted in the field so far, the actual risk and driving factors for the development of breast implant-associated tumors are yet to be clarified. BIA-ALCL and BIA-SCC are two distinct cancers arising from the breast implant capsule, with a low estimated incidence and prevalence [5,6]. The genetic mutations associated with these tumors are a key subject of interest and investigation. BIA-ALCL is the most investigated type of breast implant-associated neoplasia. Conversely, research on the genetic features underlying BIA-SCC remains scant. Beyond the ongoing research in the field, any physician should be aware of the importance of monitoring patients with breast implants for suspicious signs and symptoms and guide them to follow an adequate diagnostic work-up when a clinical suspect is present. 

The present study aims to provide a comparative and descriptive analysis of BIA-ALCL and BIA-SCC, focused on epidemiology, pathogenesis, genetic and clinical aspects, treatment, and prognosis. 

## 2. Materials and Methods

A review of the literature and official reports on breast implant-associated anaplastic large cell lymphoma (BIA-ALCL) and breast implant-associated squamous cell carcinoma (BIA-SCC) was conducted utilizing PubMed, MEDLINE, and Cochrane databases from database inception up to January 2023. English language articles pertaining to details on epidemiology, clinical presentation, pathogenesis, genetic background, disease characteristics, and disease management were included. Selected articles included original case reports, systematic reviews, and official reports from competent authorities on medical device safety. All the information collected was analyzed to provide an updated review, with the aim of comparing the current state of knowledge on BIA-ALCL and BIA-SCC. 

## 3. Results

### 3.1. Epidemiology

Over the last decade, the number of reports on BIA-ALCL has grown dramatically, and as of June 2023, a total of 1264 cases have been reported globally, including 63 deaths [5]. Among these, BIA-ALCL has been described following both aesthetic and reconstructive procedures, although data on the reason for the implant is lacking for most patients. As regards the implant type, BIA-ALCL reports have been associated with different implant types, but mostly with macrotextured ones (73% reported cases) [5]. Nonetheless, although rare, cases of BIA-ALCL in patients with smooth implants or with a history of both textured and smooth implants have been described (Table 1) [5]. The incidence and prevalence of BIA-ALCL are still uncertain due to a lack of data on breast implant surgeries [8]. The estimated Italian incidence of BIA-ALCL has shown a rising trend over the years, in parallel with increased awareness of the disease and reporting, with 3.5 per 100,000 patients receiving breast implants as of 2018 [9].

Squamous cell carcinoma of the breast implant capsule is an exceedingly rare disease, which counts only 19 reported cases worldwide and three deaths [6,10,11,12,13,14,15,16,17]. If epidemiological data on BIA-ALCL are still limited and often missing key details on the reason for implant, implant type and texture, and comprehensive patient clinical history, scientific evidence on BIA-SCC is even weaker. Based on the few reported cases, BIA-SCC seems to have a possible association with both saline and silicone implants and with both textured and smooth surfaces, without a real prevalence yet. Among the literature-reported cases, BIA-SCC cases have been described in patients who received implants for both aesthetic and reconstructive purposes [6,10,11,12,13,14,15,16,17].

### 3.2. Pathogenesis and Genetic Features

**Figure 1:**
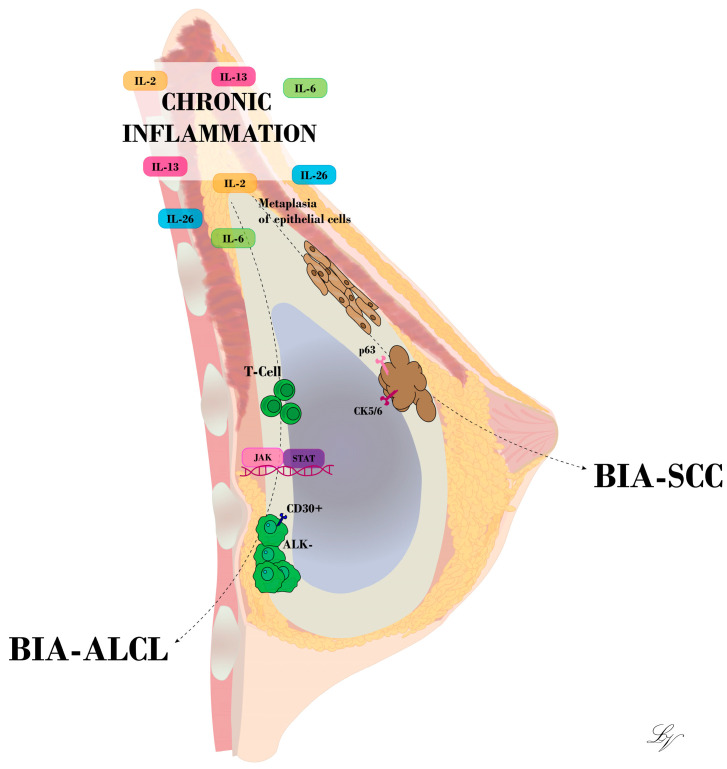
Illustration of BIA-ALCL and BIA-SCC development at the breast implant capsule. Chronic inflammation stands as a key trigger for T-cell mutation or epithelial cell metaplasia, according to the hypothesis advanced.

Experimental studies conducted in mouse models have elucidated that the implantation of alloplastic materials serves as a predisposing factor for tumorigenesis, particularly with regard to sarcomas [18]. As concerns breast implants, initial concerns about the risk of breast cancer development have been extensively disproved. Nonetheless, in recent decades, an increasing number of medical reports have shifted attention to the occurrence, although rare, of tumors arising from the breast implant capsule [19]. The implantation of devices such as breast tissue expanders or implants evokes a foreign body reaction characterized by the recruitment of immune system mediators, including macrophages and T-cells, and the formation of a fibrous tissue capsule, which envelops and isolates the implanted foreign body. Overall, this process is a benign physiological immune response led by the activation of an inflammatory cascade [20]. In the early phase, the peri-implant fibrous capsule is primarily made up of granulation tissue, which matures and is gradually replaced by collagen type I. Despite the periprosthetic tissue surrounding the implant, the chronic immune response to the implant persists, and the longer it does so, the higher the chance of DNA modification (Figure 1). Therefore, it could be inferred that the greatest risk factor for BIA-ALCL and BIA-SCC is inflammation, or more specifically, chronic inflammation. Breast cancer and a history of non-breast cancer neoplasia are not considered independent risk factors [21]. In fact, reports of these neoplasia have been found in association with both cosmetic and reconstructive breast implant capsules [22]. For BIA-ALCL, inflammatory stimuli may cause DNA modifications, which in turn triggers the activation and dysregulation of T-cells; for BIA-SCC, this may include the metaplasia of epithelial cells.

Breast implant-associated anaplastic large cell lymphoma (BIA-ALCL) is a distinct form of anaplastic large cell lymphoma (ALCL), classified as a provisional entity in the 2017 revision of the World Health Organization (WHO) classification system [23]. Recently, the 2022 WHO 5th edition Classification of Haematological Tumors (WHO HAEM5) and 2022 International Consensus Classification of Mature Lymphoid Neoplasms (22ICC) recognized breast implant-associated anaplastic large cell lymphoma (BIA-ALCL) as a definitive entity [24]. It is an uncommon anaplastic T-cell lymphoma arising from the breast implant capsule, with genetic and molecular signatures. BIA-ALCL belongs to the systemic forms of ALCL and typically shows CD30+, ALK− large anaplastic tumor cells. The tumor cells also express cytotoxic molecules, including TIA-1 and granzyme B, whereas CD3 and CD7 are always diminished or lost [25]. Although BIA-ALCL has been investigated more than BIA-SCC, its pathogenesis has yet to be clarified. As stated previously, genetic predisposition and chronic inflammation seem to be potential driving factors promoting T cell proliferation and mutation, eventually leading to lymphomagenesis [26]. The inflammation causing chronic antigenic stimulation might have different underlying triggers, among which bacterial contamination, shell shedding of particulates, shell surface friction, acellular matrices, and reactive components have been hypothesized [27,28,29,30,31]. Many emerging theories on BIA-ALCL pathogenesis are subjects of discussion and investigation. Similar to the gastric mucosa-associated lymphoid tissue B-cell lymphoma, which is caused by Helicobacter pylori-induced inflammation [21], Hu et al. (2016) found a bacterial biofilm in BIA-ALCL, suggesting that the lipopolysaccharide coat of a Gram-negative bacterium—particularly the Ralstonia pickettii, a common water-borne bacterium—could be the trigger for BIA-ALCL development [31]. Afterward, Walker and colleagues showed that Ralstonia species, identified on BIA-ALCL biofilm, are also often found in a normal periprosthetic breast capsule [32]. In a porcine model, Jacombs et al. observed that textured implants that were injected with Staphylococcus epidermidis produced an average bacterial load that was 20 times higher than that of smooth implants [33]. A correlation between the BIA-ALCL and the Ebstein–Barr Virus has also been hypothesized [34]. In recent times, there has been an increased focus on the genetics of BIA-ALCL, as understanding the alterations underlying the disease process could aid in disease prognosis and customized patient care. Germline TP53 and BRCA1/2 gene mutations have been outlined as potential risk factors [35,36,37,38,39]. The most oncogenic pathway mutation is the JAK-STAT3. The JAK-STAT pathway is an intracellular signaling that produces acute phase cytokines and proliferation of innate and adaptive constituents of the immune system: it integrates tyrosine phosphorylation on extracellular cytokine receptors to cause JAK protein phosphorylation and attracts STAT proteins to the cellular membrane for dimerization and then translocation to the nucleus to function as transcription factors [21]. Apoptosis, differentiation, and proliferation of cells are all impacted by JAK/STAT signaling. It is documented that multiple human malignancies linked to chronic immunological stimulation and inflammation also exhibit aberrant STAT3 activity [40]. Additionally, stimulation of the JAK/STAT pathway through the autocrine generation of interleukin 6 (IL-6) was demonstrated in an in vitro investigation employing BIA-ALCL-derived cell lines, indicating a potential pathogenic mechanism [41]. In 2016, Blombery et al. identified acquired activating mutations in JAK1 and STAT3 using whole-exome sequencing in two patients affected by BIA-ALCL [42]. Using targeted next-generation sequencing on seven cases of BIA-ALCL, Di Napoli et al. discovered mutations in JAK/STAT signaling pathway genes, but also in TP53 and DNMT3A [40]. The JAK/STAT pathway’s negative feedback regulator is SOCS1 (suppressor of cytokine signaling 1): mutations resulting in loss of function of SOCS1, which cause constitutive activation of JAK/STAT signaling, have been reported in both classical Hodgkin lymphomas and B-cell lymphomas [43]. A premature stop codon in SOCS1 (p.P83Rfs*20), caused by a frameshift deletion, was also found in the BIA-ALCL that carried the STAT3 mutation [40]. DNMT3A is one DNA methyltransferase needed for de novo methylation throughout the genome: according to reports, 8–22% of myeloid neoplasms and 33% of PTCLs have DNMT3A mutations, which mostly affect the enzyme’s catalytic function [44]. The discovery implies that DNMT3A mutations in BIA-ALCL may influence the methylation profile of cancerous T-cells or their progenitors, hence potentially leading to malignancy [40]. One of the most plausible causes of BIA-ALCL development is now thought to be tissue hypoxia: the hypoxia-associated biomarker carbonic anhydrase 9 (CA-9) has been dramatically upregulated in BIA-ALCL tissue compared to non-BIA ALCL tissue during RNA sequencing [45]. In one BIA-ALCL patient, the TP53 D259Y mutation was found along with a SOCS1 and STAT3 S614R mutation [40]. Patients with Li Fraumeni syndrome have been independently reported by Lee et al. and Pastorello et al. to develop BIA-ALCL [29,37]. However, TP53 mutations are comparatively uncommon in peripheral T-cell lymphomas, and it is yet unclear what role they play clinically and biologically in BIA-ALCL [25]. Only a few studies on the cytogenetic results in BIA-ALCL have been published. Lechner and colleagues created the BIA-ALCL cell lines TLBR-1, TLBR-2, and TLBR-3 and used traditional karyotyping to analyze the chromosomal aberrations in each of them [44,46]. All three cell lines have complex karyotypes: the karyotypes TLBR-2 and TLBR-3 are hypertriploid, with modal chromosome counts of 76 and 81 [44], while TLBR-1 has a modal number of 47 chromosomes [46]. Blombery et al. executed whole-genome copy number analysis on two BIA-ALCLs in a different study; one of them disclosed various copy number changes, including copy number gain of 19p and loss of 1p and 10p [42]. The deleted regions of 1p and 10p were discovered to contain the tumor suppressor genes RPL5 and GATA3, respectively. The encoded JAK family kinase TYK2, which has been shown to phosphorylate STAT1 and STAT3, raise MCL1 expression, and enhance cell survival in ALCL, was included in the focus gain of 19p [47]. Although the number of cases examined has been small, these copy number anomalies may cause or exacerbate inappropriate activation of the JAK-STAT3 pathway, necessitating more cytogenetic research. Interestingly, the activation of the JAK-STAT pathway might induce the transcription of PDL1, an immune checkpoint molecule, which engages tumor-infiltrating T lymphocytes through PD1 receptors, inducing T cell exhaustion, thus preserving a microenvironment favorable to tumor growth. Tabanelli et al. outlined in their cohort of 9 BIA-ALCL patients frequent PDL1 expression and recurrent genetic copy number alterations in PDL1. Increased PDL1 expression might result either from CNAs or be transcriptionally induced by STAT3-mediated activation. Although further studies are required, the study of the PDL1-PD1 axis in BIA-ALCL is key as it may represent a useful therapeutic target for advanced-stage disease [48,49].

Xagoraris et al. recently characterized a novel patient-derived cell line and xenograft model, the BIA-XR1 [50]. Similar to findings reported by Lechner, this cell line showed aberrant loss of T-cell markers. Cytogenetic analysis showed complex karyotypes with chromosomes X, 3, and 20 monosomy, and chromosome 21 trisomy, and multiple structural modifications (1p,5q,17p,22p). Furthermore, NGS analysis unveiled a unique feature of this cell line, the presence of KRAS mutation, which, although uncommon, might be considered for further research on new targeted therapy approaches. In the study, treatment of the BIA-XR1 cells with ERK inhibitors resulted in ERK dephosphorylation and decreased cell growth, resulting from the upregulation of CDK inhibitors and downregulation of anti-apoptotic proteins [50].

Nagel et al. recently outlined through RNA sequencing gene expression data that in contrast with ALK+ ALCL cell lines, BIA-ALCL cells show high expression of BLC2 and cyclin D2 (CCND2), the latter being a key cell cycle regulator through its activity on CDK4 and CDK6 [51]. Assessing multiple therapeutic targets, the authors showed that both inhibition of CDK4-6 through Palbociclib and inhibition of the PI3K/AKT signaling pathway through BEZ-235 enable the induction of cell cycle arrest at G1 phase, inhibiting cell proliferation, thus being relevant for further studies as therapeutic agents for BIA-ALCL [51].

The BIA-SCC is an epithelial tumor arising from cells at the implant capsule, which should be distinguished from the primary breast squamous cells carcinoma for its origin. Pathology of BIA-SCC typically shows at least one focus of SCC and squamous cells organized in nests and/or bundles, with atypical features and metaplasia [6,10,11,12,13,14,15,16,17]. BIA-SCC cells are typically positive for cytokeratin 5/6 and p63 expression at immunohistochemistry. To date, the pathogenesis of BIA-SCC remains unclear. 

The first question that arises is how a squamous cell carcinoma can differentiate from a non-epithelial tissue such as that of the periprosthetic capsule, which is predominantly populated by fibroblastic cells and immune system cells. Chronic inflammation as a cause of metaplasia and subsequent epithelial cell cancerization is among the hypothesis advanced [7]. If it is assumed that squamous cell carcinoma first arises from a benign squamous epithelium, the origin of the epithelial cells should be uncovered. One hypothesis is based on the accidental implantation of epithelial cells in the subglandular space during maneuvers such as surgical access, prosthesis implantation, or revision procedures [12]. Another origin of epithelial cells could be from the galactophore ducts, which may be severed during surgeries, implying breast implant positioning. When mammary ducts are resected, epithelial cells might remain within the implant cavity and eventually proliferate and become metaplastic upon chronic inflammatory stimuli [4]. In terms of genetics, there is currently no research for BIA-SCC available in the literature.

### 3.3. Clinical Aspects 

The clinical presentation of both BIA-ALCL and BIA-SCC is often subtle, with signs and symptoms that can be misinterpreted, causing diagnostic delays if in-depth investigation is not prompted. For both BIA-ALCL and BIA-SCC, described presentations have included breast swelling, late seroma, pain and erythema, severe capsular contracture, and skin ulceration [52,53]. For BIA-ALCL, late seroma is the most frequent presenting sign, often associated with swelling and tenderness, while the presence of a palpable mass is generally indicative of an advanced stage of the disease. Axillary lymphadenopathy, on the other hand, might be an indicator of early disease. Less frequently, BIA-ALCL might be associated with Baker IV-type capsular contracture with deformation of the breast profile and skin erythema or ulceration [30,52]. The average age at diagnosis for BIA-ALCL is around 54 years, with symptoms appearing at about 7–10 years after implantation [52]. 

Similarly, BIA-SCC may manifest with late seroma formation, breast tenderness, swelling and induration, reddening of the breast skin to erythema, and, more rarely, purulent discharge and chronic wounds [6,10,11,12,13,14,15,16,17]. In addition, capsular contracture is often appreciated and more commonly seen in BIA-SCC compared with BIA-ALCL [54]. Based on the last FDA report on BIA-SCC of March 2023, the average age at diagnosis is 53.5 years, and the average time from implantation to disease presentation is highly heterogeneous [54]. Nonetheless, considering the limited number of cases reported worldwide, further investigation is necessary for a more precise assessment of the average disease onset from implantation, presenting signs and symptoms, owing to the possibility of symptoms misinterpretation and diagnostic delay, which is likely given the rarity of the disease and the lack of information at the time of the first diagnoses made.

### 3.4. Disease Diagnosis and Management 

For BIA-ALCL, consensus recommendations for the diagnostic and treatment workup have been drafted based on current evidence [52]. Consensus recommendations for BIA-ALCL diagnosis, which include a tailored diagnostic work-up, are key considering the subtle clinical presentation, which may result in diagnostic delay and disease progression. Indeed, as described above, common presenting signs and symptoms are not disease-specific. In the presence of any of the signs/symptoms typically associated with the disease, a breast ultrasound examination is indicated. Following the performance of breast US, if periprosthetic fluid is appreciated, ultrasound-guided needle aspiration of the fluid should be performed, with cytologic and immunohistochemical analysis, which might show typical BIA-ALCL cell signatures [52]. If the effusion is more than 20 mL, a part must be sent to the microbiology laboratory for a cultural examination and differential diagnosis. If there is evidence of lymphadenomegaly, a lymph node biopsy with histologic examination and immunohistochemical analysis is necessary, while if there is evidence of a mass, a breast MRI is requested, and then mass resection with histologic and immunohistochemical analysis is performed. Instead, if there is Baker type IV capsular contracture associated with seroma or locoregional lymphadenomegaly, a complete capsulectomy and histologic and immunohistochemical analysis are performed. A confirmed diagnosis of BIA-ALCL is made on fluid, periprosthetic capsule, mass, or lymph node, showing evidence of greater than 10% CD30+ cellularity, ALK− and atypical anaplastic large cell morphology. As a BIA-ALCL diagnosis is made, a PET-CT scan is required for disease staging and treatment planning [52]. For any disease stage, implant removal and en-bloc capsulectomy are the gold standard treatments. In the presence of lymph node infiltration or distant metastasis, chemotherapy, immunotherapy, or radiation therapy can also be performed as appropriate. Based on current reports, the mortality rate is about 2.8% after one year [55]. 

For BIA-SCC, there are no well-defined guidelines for the diagnostic work-up and management. Adding complexity to diagnosis, its clinical presentation often overlaps with that of BIA-ALCL, and prior to advancing the hypothesis of a BIA-SCC, it is crucial to rule out a primary breast squamous cell carcinoma (SCC). Ultimately, considering that the clinics of primary SCC of the breast are similar to that of BIA-SCC, a definitive diagnosis depends on histological evaluation [4]. As recommended by the Italian Competent Authority and working group “Late Periprosthetic Fluid Collection after Breast Implant”, in any patient with late periprosthetic fluid collection detected by US examination, fine needle aspiration of the fluid and cytological analysis should be performed [56]. The ascertained cases of BIA-SCC reported in the literature show positive flow cytometry for squamous cells and keratin and positive CK 5/6 and p63 at immunohistochemistry [10,11,12,13,14,15,16,17]. Further investigation with breast MRI can confirm the periprosthetic effusion and unveil the presence of lesions that form a pericapsular mass first. A PET-CT scan is performed to assess the presence of metastases and stage the disease. Indeed, many of the patients diagnosed with BIA-SCC present extracapsular invasion at the time of diagnosis. The metastatic sites include the axilla, upper and lower limbs, soft tissues, liver, lung, kidney, and meninges [11,57]. Based on the reported cases, the mortality rate at six months is 48% [54]. Management of the reported cases of BIA-SCC involved implant removal with capsulectomy. In some patients with breast tissue invasion, mastectomy, and axillary dissection were necessary immediately or performed as a second-stage procedure. Neoadjuvant and adjuvant chemotherapy and radiotherapy have also been used in patients with extracapsular disease, although with limited effects [10,15,16,57,58].

**Table 1:** Summary of current updated evidence on breast implant-associated neoplasia (BIA-ALCL and BIA-SCC).

	BIA-ALCL	BIA-SCC
*Tumor characterization*	Non-Hodgkin T-cell anaplastic large lymphoma arising from the breast implant capsule	Squamous cell carcinoma (SCC) arising from the breast implant capsule. It should be distinguished from primary breast SCC.
*Known cases* [5,6]	1264	19
*Mean age at diagnosis (Median)* [5,6]	54	53.5
*Diagnosis following implantation (years range)* [5,6]	0–40	7–42
*Implant type* [5,6]	Silicone and Saline implants	Silicone and Saline implants
*Association with breast reconstruction* [5,6]	Yes	Yes
*Association with breast augmentation* [5,6]	Yes	Yes
*Implant texture* [5,6]	Most cases have been associated with textured implants (73%). To date, an association with smooth implants cannot be excluded.	Textured implants Smooth implants
*Clinical presentations* [10,11,12,13,14,15,16,17,52,53,57]	Late seroma Breast swelling Pain Erythema Capsular contracture Lymphadenopathy Mass/lump	Late seroma Breast swelling Pain Erythema Capsular contracture Lymphadenopathy Mass/lump
*Genetic susceptibility* [29,36,37]	BIA-ALCL cases have been reported in patients with TP53 and BRCA1/2 mutations. Further investigation is required to define the associated risk.	Unknown
*Tumour genetics* [40,41,42,50]	Several mutations have been identified (JAK1, STAT3, SOCS1, TP53, DNMT3A, K-RAS)	Unknown
*Diagnostic assessment* [52]	If seroma is detected via US, US-guided FNA is recommended. Other imaging studies are performed based on clinical presentation (MRI); a biopsy is recommended in the presence of a mass. PET/CT is recommended for staging.	If seroma is detected via US, US-guided FNA is recommended. Other imaging studies are performed based on clinical presentation (MRI); a biopsy is recommended in the presence of a mass. PET/CT is recommended for staging.
*Tumour cell characterization* [54]	Flow cytometry + for T-cells; CD30+; ALK−.	Flow cytometry + for squamous cells and keratin; CK 5/6+; p63+.
*Treatment* [10,11,15,52,57,58]	For localized disease (Stages I-IIA), implant removal with en-bloc capsulectomy is recommended. For extracapsular disease (IIB-IV), implant removal with en-bloc capsulectomy is recommended. A multidisciplinary evaluation is required to evaluate adjuvant/neoadjuvant chemotherapy/immunotherapy and adjuvant radiotherapy.	Treatment recommendations are limited. En-bloc capsulectomy with implant removal is ordinarily performed. Adjuvant chemotherapy and radiotherapy have been utilized.
*Reported mortality* [54]	2.8% at one year	43.8% at 6 months

## 4. Discussion

Over the last decade, the BIA-ALCL and BIA-SCC have emerged as late neoplastic complications associated with the use of breast implants and arising from the periprosthetic capsule, a physiological tissue from an immunological response to the implant. The estimates for the incidence suggest that BIA-ALCL is uncommon, and BIA-SCC is a rather rare complication of breast implants. Current data show a similar average age at presentation. In contrast, the time from implant placement to onset of signs and symptoms of disease seem to be higher for BIA-SCC. However, the paucity of cases of BIA-SCC reported worldwide, as the disease is rare, might pose limits to symptoms misinterpretation and diagnostic delay. As concerns the implant type, most BIA-ALCL cases have been associated with macrotextured implants. In contrast, current data on BIA-SCC cases do not show a significant difference between smooth and textured implants. For both tumors, further investigation of the pathogenetic mechanisms is required. Nonetheless, chronic inflammatory stimuli and genetic factors have been hypothesized as potential driving factors for tumorigenesis. While in the BIA-ALCL, chronic stimuli drive lymphomagenesis, acting on the population of T lymphocytes recruited at the implant site, in the BIA-SCC epithelial cells undergoing metaplastic changes and subsequent cancerization seem to be at the core of the tumor development process. However, since there are no epithelial cells in the periprosthetic tissue, these would come from either external contamination or the galactophore duct epithelial cells that were resected during the reconstruction or implantation procedures. Because periareolar access has a larger chance of causing a galactophore recision than inframammary fold access, it is imperative that we exercise caution when manipulating the implant and avoid causing friction with the skin surface [59,60,61,62]. Despite being two distinct tumor types with different behaviors, BIA-ALCL and BIA-SCC share similar signs and symptoms of presentation, including late seroma, breast tenderness, and swelling, which require prompt investigation with an ultrasound exam and consequential cytological examination if the presence of periprosthetic fluid is detected. To date, despite the published recommendations that guide the diagnostic work-up and treatment of BIA-ALCL, the clinical framing of BIA-SCC is still uncertain [52]. As regards imaging, US coupled with US-guided FNA is the first-line examination for both BIA-SCC and BIA-ALCL. Cytology and immunohistochemistry, including CD30, ALK, CK 5/6, p63, and flow cytometry to search for T-cells, squamous cells, and keratin, are key to the differential diagnosis. Further investigation with MRI is required in the cases of suspected implant rupture and to outline the presence of periprosthetic masses [63,64]. PEC-CT evaluation is key to assessing the presence of distant metastasis and disease staging in both tumors [65,66]. Mammography is not very useful in these circumstances, but it might be when the patient presents early with a mass [67]. It would be interesting to promote new technologies, such as AI-based medical systems, for accurate and early diagnosis by evaluating asymmetries in shape and volume of images since mammography is used as a screening tool for breast cancer [D]. R. Bayareh-Mancilla and colleagues have suggested a mammography method based on skin thickness measured using Growing Region Seed (GRS) and Dynamic Time Warping (DTW): in comparison to current operator-dependent systems, these represent crucial components that could enhance AI- models and encourage more precise and early screening [68].

While BIA-ALCL has an indolent course and good prognosis at the early stages of diagnosis, the BIA-SCC seems to be more aggressive, comparable with that of primary squamous cell carcinomas, with higher rates of extracapsular invasion at the time of diagnosis, and mortality. For BIA-ALCL, there has only been one documented instance of spontaneous regression [43]. When either BIA-ALCL or BIA-SCC are diagnosed, implant removal with en-bloc capsulectomy is imperative. Chemotherapy and radiation therapy have been utilized in the management of advanced-stage BIA-SCC, although the benefits of these approaches are yet to be confirmed due to the lack of guidelines for treating metastatic BIA-SCC. Given the complexity of etiology, research on genetic traits may be useful for both prevention and treatment. JAK-STAT pathway activation is characteristic of BIA-ALCL: it is primarily caused by point mutations in JAK1 and STAT3. Therefore, unlike other forms of ALCL, BIA-ALCL exhibits a more consistent molecular signature in addition to its distinctive clinical manifestations that are linked to a breast prosthesis. JAK-STAT may be a viable therapeutic target in situations in case of severe systemic illness; however, to date, evidence is still scant, and surgical treatment remains the mainstay of treatment [42]. JAK-STAT inhibition has been shown to happen in TLBR cell lines by Lechner et al. [44]. When sunitinib, a JAK-STAT inhibitor, was given in vivo to encourage tumor cell death, a dose-dependent effect was demonstrated [25]. The Food and Drug Administration has approved the following list of potential JAK-STAT3 pathway treatments: fedratinib, ruxolitinib, tofacitinib, baricitinib, upacitinib, atovaquone, pyrumethamine, cetuximab, and pimozide [21]. A greater understanding of the genetic, functional, and potential clinical aspects of the activated JAKSTAT3 phenotype and possible PD-L1 expression in BIA-ALCL is necessary, given the recent advancements in JAK inhibitors and antibodies that block the PD1/PD-L1 axis [46,48]. Further areas of investigation for new therapeutic strategies might involve the inhibition of MEK-ERK kinases, PI3K/AKT, and CDK4-6, as suggested by recent findings [48,49,50,51].

It is unknown how genetically comparable BIA-SCC is to cutaneous or primary breast squamous cell carcinoma. Currently, there is not enough information available in the literature to establish a framework for this tumor’s genetic characterization.

## 5. Conclusions

BIA-ALCL and BIA-SCC have emerged as two uncommon complications associated with breast implants. Despite being two different tumors, they both exhibit the most common warning signals, which include breast swelling, discomfort, and late seroma. The hypothesis that abnormal activation of the JAK-STAT3 signaling pathway plays a role in the genesis and evolution of BIA-ALCL tumors has been strengthened by recent genetic molecular research. These findings, along with efforts made to characterize the tumor’s genetic and molecular hallmark alterations, have paved the way for the study of novel therapeutic targets, which might be key in the future. More thorough oncogenomic research is, therefore, necessary to clarify the BIA-ALCL genetic landscape, the frequency of JAK-STAT3 pathway mutations, and their functional implications. For BIA-SCC, the pathogenic landscape is still underexplored, and a deeper understanding of the disease is required for the development of more evidence-based guidelines for diagnosis and treatment strategies. For suspected BIA-SCC cases, as presented in the literature, the diagnostic approach used is similar to that of BIA-ALCL, although the clinical course is different. Time and further research in the field are required to define better the actual risk of development of BIA-ALCL and BIA-SCC among patients with breast implants, the pathogenetic mechanisms, and how different implant types may affect the occurrence of such diseases. Meanwhile, any physician should be aware of these diseases, monitor suspicious signs and symptoms, guide patients when clinical suspects are present, and follow the best diagnostic and treatment work-up according to the actual knowledge in the field.

## Data Availability

No new data were created or analyzed in this study. Data sharing is not applicable to this article.
